# Self-Assembled Nanoparticles Based on Block-Copolymers of Poly(2-Deoxy-2-methacrylamido-d-glucose)/Poly(*N*-Vinyl Succinamic Acid) with Poly(*O*-Cholesteryl Methacrylate) for Delivery of Hydrophobic Drugs

**DOI:** 10.3390/ijms222111457

**Published:** 2021-10-24

**Authors:** Mariia Levit, Alena Vdovchenko, Apollinariia Dzhuzha, Natalia Zashikhina, Elena Katernyuk, Alexey Gostev, Eugene Sivtsov, Antonina Lavrentieva, Tatiana Tennikova, Evgenia Korzhikova-Vlakh

**Affiliations:** 1 Institute of Macromolecular Compounds, Russian Academy of Sciences, Bolshoy pr. 31, 199004 St. Petersburg, Russia; musia_1@yahoo.com (M.L.); polinadzhuzha@mail.ru (A.D.); nzashihina@bk.ru (N.Z.); st068478@student.spbu.ru (E.K.); pjeka@yahoo.fr (E.S.); 2 Institute of Chemistry, Saint-Petersburg State University, Universitetskiy pr. 26, 198504 St. Petersburg, Russia; vdovchjenko@yandex.ru (A.V.); tennikova@mail.ru (T.T.); 3 Saint-Petersburg State Institute of Technology, Technical University, Moskovskiy pr. 26, 190013 St. Petersburg, Russia; ga81@rambler.ru; 4 Institute of Technical Chemistry, Gottfried-Wilhelm-Leibniz University of Hannover, 30167 Hannover, Germany; lavrentieva@iftc.uni-hannover.de

**Keywords:** amphiphilic copolymers, block-copolymers, bio-inspired copolymers, controlled radical polymerization, polymer nanoparticles, drug delivery systems, paclitaxel delivery

## Abstract

The self-assembly of amphiphilic block-copolymers is a convenient way to obtain soft nanomaterials of different morphology and scale. In turn, the use of a biomimetic approach makes it possible to synthesize polymers with fragments similar to natural macromolecules but more resistant to biodegradation. In this study, we synthesized the novel bio-inspired amphiphilic block-copolymers consisting of poly(*N*-methacrylamido-d-glucose) or poly(*N*-vinyl succinamic acid) as a hydrophilic fragment and poly(*O*-cholesteryl methacrylate) as a hydrophobic fragment. Block-copolymers were synthesized by radical addition–fragmentation chain-transfer (RAFT) polymerization using dithiobenzoate or trithiocarbonate chain-transfer agent depending on the first monomer, further forming the hydrophilic block. Both homopolymers and copolymers were characterized by ^1^H NMR and Fourier transform infrared spectroscopy, as well as thermogravimetric analysis. The obtained copolymers had low dispersity (1.05–1.37) and molecular weights in the range of ~13,000–32,000. The amphiphilic copolymers demonstrated enhanced thermal stability in comparison with hydrophilic precursors. According to dynamic light scattering and nanoparticle tracking analysis, the obtained amphiphilic copolymers were able to self-assemble in aqueous media into nanoparticles with a hydrodynamic diameter of approximately 200 nm. An investigation of nanoparticles by transmission electron microscopy revealed their spherical shape. The obtained nanoparticles did not demonstrate cytotoxicity against human embryonic kidney (HEK293) and bronchial epithelial (BEAS-2B) cells, and they were characterized by a low uptake by macrophages in vitro. Paclitaxel loaded into the developed polymer nanoparticles retained biological activity against lung adenocarcinoma epithelial cells (A549).

## 1. Introduction

Over the last decade, attention to nanomaterials based on self-assembling amphiphilic polymers has been steadily growing [1,2]. In particular, self-assembled nanometer-sized polymer particles have proven to be promising objects for creating drug delivery and detection systems [3]. Compared to other nanomaterials, such as liposomes, lipid nanoparticles, carbon nanotubes, quantum dots, and others, polymer nanoparticles have an undeniable advantage—a high variability of resulting structures and properties. The selection of the chemical structure of macromolecules enables the preparation of nanoparticles with specified characteristics, i.e., size, morphology, stability, cellular uptake, drug release kinetics, etc.

Among many types of copolymers used for the construction of drug delivery systems, amphiphilic block-copolymers stand out due to their potential for self-assembly [4,5]. In particular, amphiphilic macromolecules are able to accumulate at the boundary of two phases. In aqueous solutions, the orientation of macromolecules occurs in such a way that hydrophobic sites tend to minimize the contact with an aqueous environment to achieve a minimum energy. With an increase in the concentration of the amphiphilic copolymer in solution, the free energy of the system increases due to unfavorable interactions between water molecules and the hydrophobic fragment of the macromolecule. This leads to the self-induced structuring of polymer macromolecules to decrease the entropy of the system. As a result, the formation of spherical or cylindrical polymer micelles, as well as polymer vesicles or polymersomes, is possible [5]. All these nanoparticles are suitable for encapsulation of hydrophobic and amphiphilic drugs [6,7,8,9].

The biocompatibility of nanoparticles considered as drug delivery systems is one of the most important requirements, as this determines the applicability of such systems in vivo [10]. One of the ways to increase the biocompatibility of polymer particles is the use of natural macromolecules, such as heparin, chitosan, chondroitin sulfate, collagen, etc. [11]. Although these polymers are biocompatible, the high rate of biodegradation of some natural polymers during enzymatic, oxidative, and hydrolytic processes in the body significantly reduces the lifetime of delivery systems [12]. Another way to increase the biocompatibility of polymer systems in vivo is the application of synthetic bio-inspired macromolecules [13]. They can be fully biodegradable, such as poly(lactic acid) [14] or poly(L-amino acids) [8], or possess non-biodegradable carbon–carbon backbone bearing cleavable bio-inspired side units, such as saccharide fragments [15]. In order to increase the lifetime of delivery systems based on amphiphilic copolymers, the hybrid systems that combine biodegradable and non-biodegradable polymer fragments can be a matter of choice [16,17]. Currently, a number of amphiphilic hybrid and fully synthetic copolymers have been synthesized. Poly(ethylene glycol) (PEG), polyoxazolines, different polysaccharides, hydrophilic poly(amino acids), and synthetic glycopolymers are usually used as hydrophilic blocks, while aliphatic polyesters (poly(lactic acid) (PLA), polycaprolactone, etc.), polycarbonates, hydrophobic poly(amino acids), polystyrene, poly(butyl methacrylate), etc., are utilized as hydrophobic blocks [17,18,19,20,21,22]. 

In the present study, we focused on (i) the synthesis of novel amphiphilic block-copolymers containing non-biodegradable carbon–carbon backbone and bio-inspired side units, (ii) the preparation and characterization of nanoparticles based on these copolymers, as well as (iii) their biological evaluation. Poly(2-deoxy-2-methacrylamido-d-glucose) (PMAG) and poly(*N*-vinyl succinamic acid) (PVSAA) were selected as hydrophilic blocks. PMAG belongs to the class of synthetic glycopolymers possessing d-glucose side units linked with a non-biodegradable main chain via amide bonds. It is known that synthetic glycopolymers are hydrophilic and biocompatible macromolecules [23,24]. Moreover, glycopolymers with non-ionizable saccharide units can diminish the uptake of nanoparticles by macrophages, which is important for an enhanced lifetime of drug delivery systems in vivo. Furthermore, the partial oxidation of the vicinal hydroxyls of saccharide units provides the formation of highly reactive aldehydes, which are very useful for conjugation with amino-bearing biomolecules or labels [25]. Another hydrophilic block, namely PVSAA, represents a polymer bearing the half amide of succinic acid as side moieties. Similar to d-glucose in PMAG, succinic acid is also a natural compound whose polymer is expected to be biocompatible. Unlike PMAG, which is a neutral hydrophilic polymer, PVSAA is negatively charged. To the best of our knowledge, PVSAA has not been previously described for the synthesis of amphiphilic copolymers and has not been tested in biological experiments.

Poly(*O*-cholesteryl methacrylate) (PChMA) was selected as a hydrophobic fragment. In contrast to d-glucose and succinic acid, the cholesterol units are bound to the main chain via ester bonds. It is known that cholesterol is one of the components of cell membranes that provide their rigidity, and it is involved in many vital processes. Furthermore, cholesterol favors the biocompatibility of a delivery system and can cause better permeability of polymer particles into cells [26]. For example, Sevimli et al. reported the synthesis of poly(methacrylic acid)-*b*-poly(O-cholesteryl methacrylate). The authors revealed that the use of a cholesterol-containing copolymer improved cell penetration by 20–25% [27]. 

Considering that the selected bio-inspired copolymers have non-biodegradable main chains, they are subject to requirements of the molecular weight of synthetic polymers. The upper limit of molecular weight for the renal filtration of non-biodegradable copolymers is 30,000–50,000. This depends on the polymer’s nature, shape, molecular conformation, and flexibility [28]. Since hydrophobic cholesterol is bound to the main chain by an ester bond, it can undergo hydrolysis over time, which, in turn, will provide the liberation of carboxyl groups in the copolymer. As a result, the greater hydrophilization and better solubility of the copolymers may favor their enhanced removal from the body. 

Due to the molecular weight limitation for non-biodegradable biomedical copolymers, they must have a low dispersity. In this regard, the methods of controlled polymerization, which have been widely developed over the last two decades, are very effective for synthesizing well-defined polymers [29,30,31]. The block-copolymers of interest can be efficiently synthesized by the controlled, or so-called “living”, radical polymerization. Radical addition–fragmentation chain-transfer (RAFT) polymerization is known for its suitability for the polymerization of a wide range of monomers in comparison with other methods of radical polymerization. Monomers such as (meth)acrylates, (meth)acrylamides, styrene derivatives, butadiene, vinyl acetate, N-vinylpyrrolidone, etc., can be successfully polymerized with the use of the RAFT technique [32,33,34]. Furthermore, a wide variety of structures can be used in the side chain, including covalently bound natural molecules such as saccharides, amino acids, or cholesterol [35,36,37,38]. Moreover, RAFT polymerization is widely used for the synthesis of block-copolymers where a homopolymer, formed during the first step, serves as a macro-RAFT agent for the synthesis of the second block from another monomer [39]. Recently, the successful syntheses of well-defined glycopolymers [24,40] and their copolymers with poly(diethylene glycol methacrylate) [35], azobenzene methacrylate [41], *N*-(2-aminoethyl)methacrylamide [42], etc., by RAFT polymerization or a combination of RAFT with other polymerization techniques, such as ring-opening polymerization [17,43] or click reactions [23], were reported. 

In this study, the novel bio-inspired block-copolymers, containing PMAG and PVSAA as hydrophilic blocks and PChMA as a hydrophobic block, were synthesized by RAFT polymerization and carefully characterized to evaluate their molecular weights, composition, and thermal stability. The obtained copolymers possessed the ability to self-assemble in aqueous media into nanometer-sized structures. The characteristics and morphology of the self-assembled nanoparticles, as well as their cytotoxicity and uptake by macrophages, were investigated. In addition, the efficiency of developed polymer nanoparticles as paclitaxel delivery systems was determined as half-maximal inhibition concentrations (IC_50_) with the use of lung adenocarcinoma epithelial cells (A549). 

## 2. Results and Discussion

The synthesis of well-defined polymers by RAFT polymerization is limited by the selection of a suitable chain-transfer agent (CTA) as well as a solvent capable of dissolving both the monomer and the resulting product. Essentially, the used chain-transfer agents (CTAs) include dithioesters (DTEs) [33,44], trithiocarbonates (TTCs) [45], dithiocarbamates [46], and xanthates [47], which mediate polymerization via a reversible chain-transfer process. The efficiency of CTA depends on both the monomer and the properties of the leaving groups (R) and stabilizing groups (Z) of CTA [48]. 

### 2.1. Synthesis of PMAG-b-PChMA

In general, the synthesis of a diblock-copolymer by RAFT polymerization includes a synthesis of a homopolymer, which further serves as a macro-RAFT agent in RAFT copolymerization with another monomer. In this case, it should be considered that the stabilizing Z group must ensure high control over the polymerization for both monomers, otherwise an increase in the dispersity for one of the polymer blocks would be observed [49,50]. Moreover, the order of synthesis of the polymer blocks plays a significant role because the first block serves as a macro-R group in the polymerization of the second monomer. In this context, the macro-R group must be a good leaving group relative to the second monomer and efficiently reinitiate its polymerization. 

In our case, 2-deoxy-2-methacrylamido-d-glucose (MAG) and *O*-cholesteryl methacrylate (ChMA) are methacrylamide and methacrylate types of monomers. For these monomers, dithiobenzoates (DTBs) demonstrate excellent control over molecular weight characteristics as well as a low retardation, but they are prone to hydrolysis in aqueous media [44,49]. Thus, polymerization in an organic medium (usually *N*,*N*-dimethylformamide (DMF)) with the use of this kind of CTA is preferable. In the presence of DTB, the activity of the leaving R group is similar for both methacrylate and methacrylamide polymerization. It means that control over the molecular weight characteristics of block-copolymers can be maintained regardless of the order of homopolymer synthesis [50]. Despite this, PMAG and PChMA have a huge difference in their solubility, and the polymerization of MAG using PChMA as a macro-RAFT would be more difficult. In this regard, the synthesis of PMAG-*b*-PChMA was carried out by polymerization of ChMA on PMAG-DTB, using the latter as a macro-RAFT agent. 

**Figure 1:**
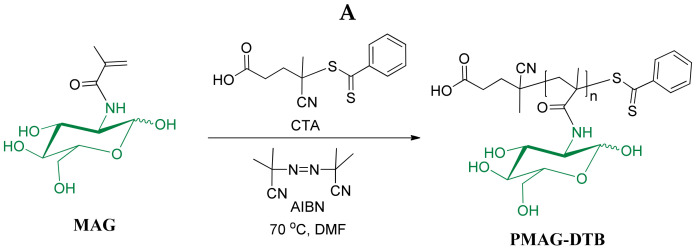

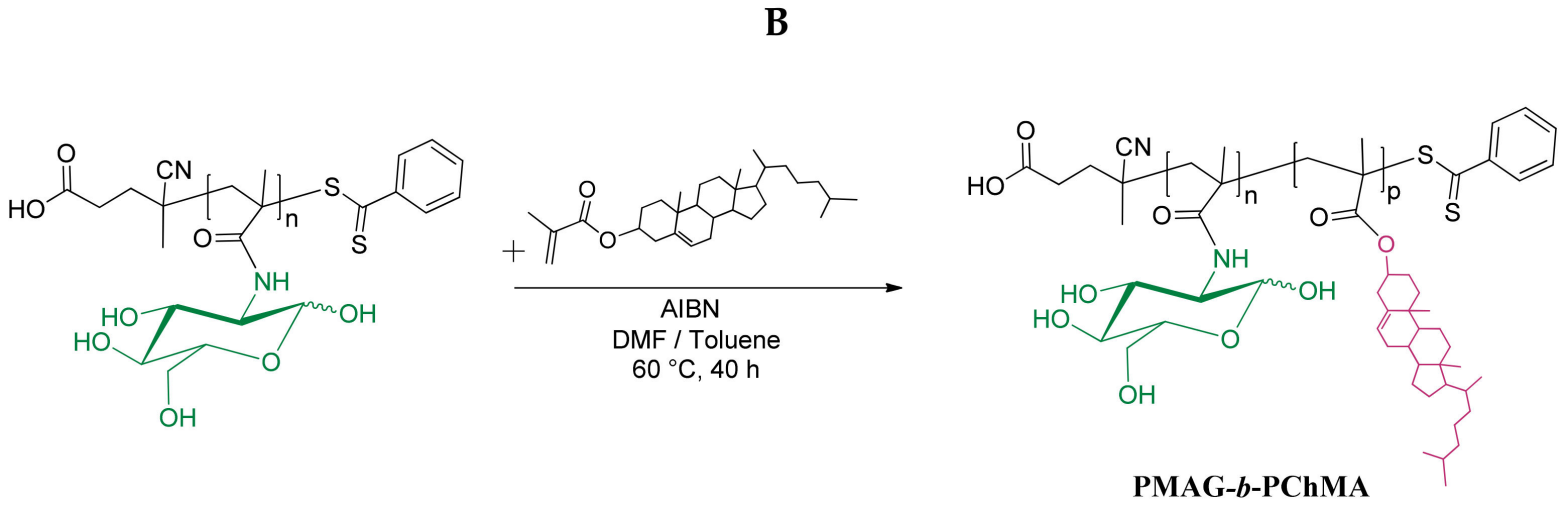
Schemes of PMAG (**A**) and PMAG-*b*-PChMA (**B**) synthesis.

PMAG-DTB was synthesized by RAFT polymerization using 4-cyanopentanoic acid-4-dithiobenzoate as CTA, using the protocol developed in our group earlier [24]. The polymerization process was carried out at 70 °C with continuous stirring for 16 h using the following molar ratio of reagents: [MAG]:[DTB]:[AIBN] = 20:1:0.25. The scheme and conditions for the synthesis of PMAG-DTB are shown in Figure 1A. The molecular weight characteristics, determined by size-exclusion chromatography (SEC) with triple detection, are presented in Table 1 (sample #1). The structure of the homopolymer obtained was testified by ^1^H NMR spectroscopy (Figure S1, Supplementary Materials). 

The scheme for the synthesis of PMAG-*b*-PChMA is shown in Figure 1B. The polymerization process was carried out at 60 °C with continuous stirring for 40 h using the following molar ratio of reagents: [ChMA]: [PMAG-DTB]:[AIBN] = 18:1:0.35. Due to the fact that the resulting copolymer was poorly soluble in organic and aqueous solvents, it was impossible to determine its composition and molecular weight by ^1^H NMR spectroscopy and SEC. The characterization of this copolymer was carried out by Fourier transform infrared (FTIR) spectroscopy (Figure S2). 

**Figure 2:**
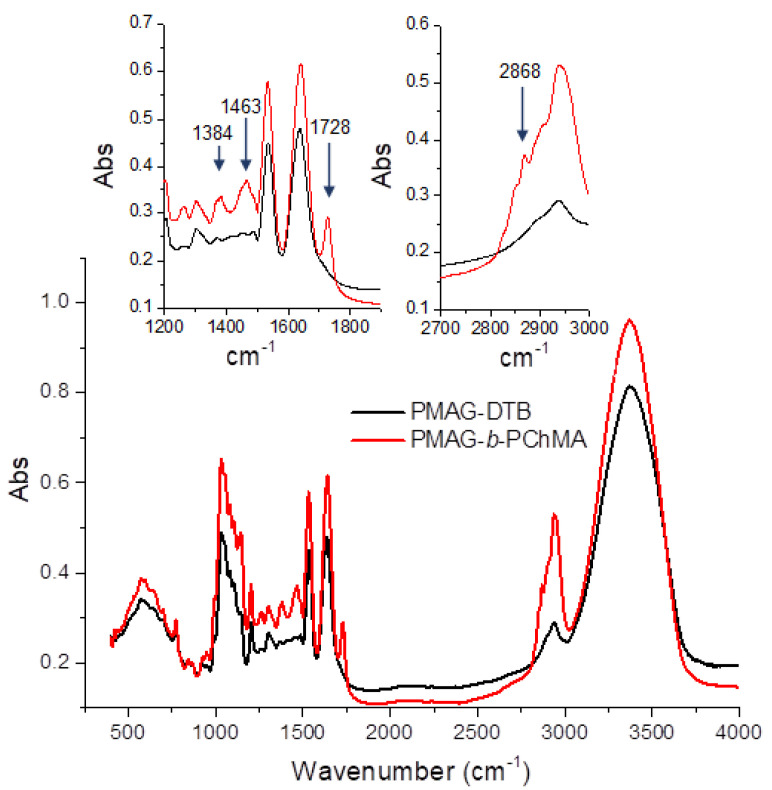
FTIR spectra of PMAG-DTB and its copolymer with PChMA.

As seen from Figure 2, a characteristic band at 1728 cm^−1^, corresponding to the valence vibrations of the C=O bond in esters, appears in the FTIR spectrum registered after copolymerization. In addition, the appearance of the characteristic bands at 2868, 1463, and 1384 cm^−1^ indicates the presence of valence symmetric, deformation asymmetric, and symmetric vibrations of the CH_3_ groups, presenting in a much larger number in PChMA in comparison with PMAG-DTB.

The content of ChMA in the PMAG-*b*-PChMA was established by FTIR spectroscopy using a calibration plot built for a mixture of PMAG and PChMA homopolymers (Figure S2, Supplementary Materials). This approach was earlier reported in several papers for the determination of the composition for block-copolymers [51,52]. The ratio of the intensities of the absorption bands specific to PMAG and PChMA was used to determine the molar fraction of the hydrophobic block (PChMA) in PMAG-*b*-PChMA. Based on the obtained data and the molecular weight of PMAG (determined by SEC with triple detection), the number of ChMA units in the copolymer and the number average molecular weight (*M_n_*) of the PChMA block were found to be 18 and 8100, respectively.

### 2.2. Synthesis of PVSAA-b-PChMA-b-PVSAA

In order to prepare the block-copolymers containing a half amide of succinic acid in the side chain of the hydrophilic block, poly(*N*-vinylsuccinimide)-containing block-copolymers were initially synthesized as precursors. In this case, *N*-vinylsuccinimide (VSI) and ChMA were used as monomers, while *S*,*S*-dibenzyl trithiocarbonate was selected as CTA. It is known that symmetrical TTCs have two homolytic leaving groups and, consequently, after polymerization, the TTC function remains in the middle of the formed polymer. At the next copolymerization step, TTC causes the insertion of the second block in the middle of polymer chain, forming the ABA triblock-copolymer [53,54,55,56]. 

**Figure 3:**
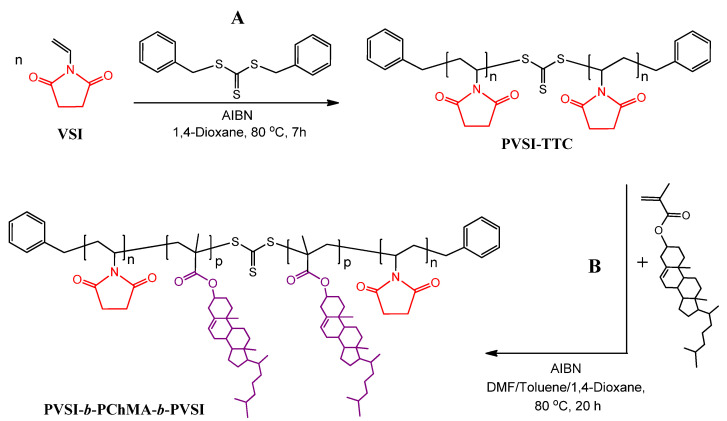
Schemes of synthesis of PVSI (**A**) and PVSI-*b*-PChMA-*b*-PVSI (**B**).

TTCs are less active CTAs in comparison with DTB [57]. In turn, they are able to provide good control over the polymerization of the vinyl monomers [55,58] and methacrylates [54], and at the same time provide low retardation. Furthermore, they are more hydrolytically stable than DTB [59]. Polymethacrylates are among the best leaving groups and are significantly more effective as an R group when compared to *N*-vinylpyrrolidone or *N*-vinylsuccinimide [50,60]. Considering this, the PVSI homopolymer was synthesized first, while the copolymerization with ChMA was carried out at the second step to obtain their block-copolymer. The scheme of synthesis of the PVSI-containing precursor copolymer is illustrated in Figure 3. 

At the first step, PVSI-TTC samples of two different molecular weights were synthesized. The obtained PVSI homopolymers were characterized by SEC. The molecular weight characteristics of PVSI-TTC used as a macro-RAFT agent for the polymerization of ChMA are provided in Table 1 (samples #2 and #3). Both samples had a rather narrow dispersity and differed in their molecular weights. 

PVSI-TTC samples were applied as the macro-RAFT agents for the polymerization of ChMA. The ratio between ChMA, macro-RAFT agent, and initiator was constant in all cases and was the following: [PVSI-TTC]:[ChMA]:[AIBN] = 1:20:0.4. The synthesis of block-copolymer was carried out at 80 °C for 20 h. The obtained block-copolymers were analyzed by FTIR and ^1^H NMR spectroscopy (see Section 3.3.2). The relative content of PChMA in the copolymer was determined by ^1^H NMR spectroscopy using the ratio of the integral signal intensities at 5.3–5.4 and 3.3–3.5 ppm, which are characteristic signals for the protons of H–C=C in PChMA and H–C–N in PVSI, respectively (Figure S3, Supplementary Materials). The compositions of block-copolymers obtained are presented in Table 1 (samples #2 and #3). 

**Figure 4:**
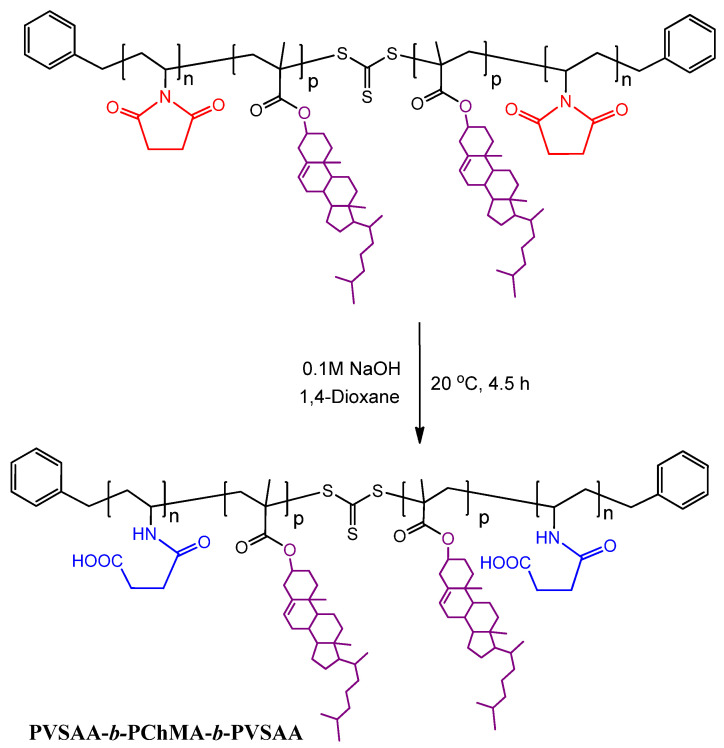
Scheme for PVSAA-*b*-PChMA-*b*-PVSAA preparation.

At this stage, the obtained PVSI-*b*-PChMA-*b*-PVSI was hydrophobic. To convert it to amphiphilic structure, the alkaline hydrolysis under mild conditions was performed (Figure 4). The applied approach is known to provide the selective disclosure of the succinimide cycle in the presence of most other comonomers, including (meth)acrylates [61]. As the initial PVSI fragments were of different lengths, the amphiphilic block-copolymers with different lengths of the hydrophilic fragments were obtained (further samples #h2 and #h3) as a result of succinimide cycle hydrolysis. 

**Figure 5:**
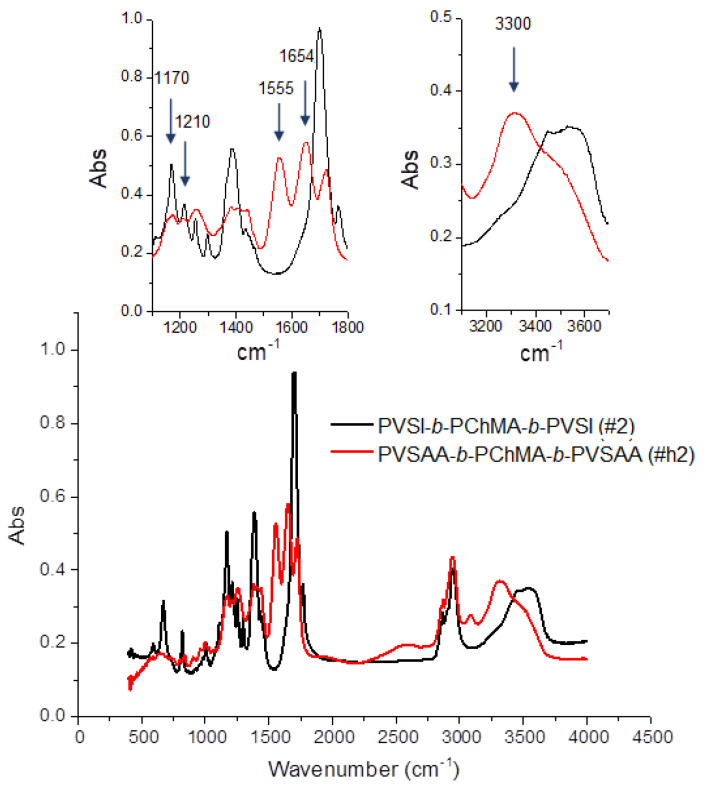
FTIR spectra of PVSI-*b*-PChMA-*b*-PVSI and PVSAA-*b*-PChMA-*b*-PVSAA.

The appearance of the characteristic bands in the FTIR spectrum at 1555, 1654, and 3300 cm^−1^, corresponding to valence vibrations of N–C=O (amide II), C=O (amide I), and N–H groups, respectively, as well as the disappearance of bands at 1170 and 1210 cm^−1^, corresponding to C–N skeletal vibrations of succinimide ring, confirmed its disclosure in PVSI (Figure 5). At the same time, the bands characteristic of PChMA remained. In particular, the bands at 1258 and 1725 cm^−1^, corresponding to valence vibrations of O-C-C and ester C=O groups, as well as at 2942, 2868 and 1435 cm^−1^ characteristic to asymmetric and symmetric stretching, and deformation vibrations of CH_2_ groups, which are abundant in PChMA, were detected in the modified copolymer.

### 2.3. Thermal Properties of Synthesized Block-Copolymers

**Figure 6:**
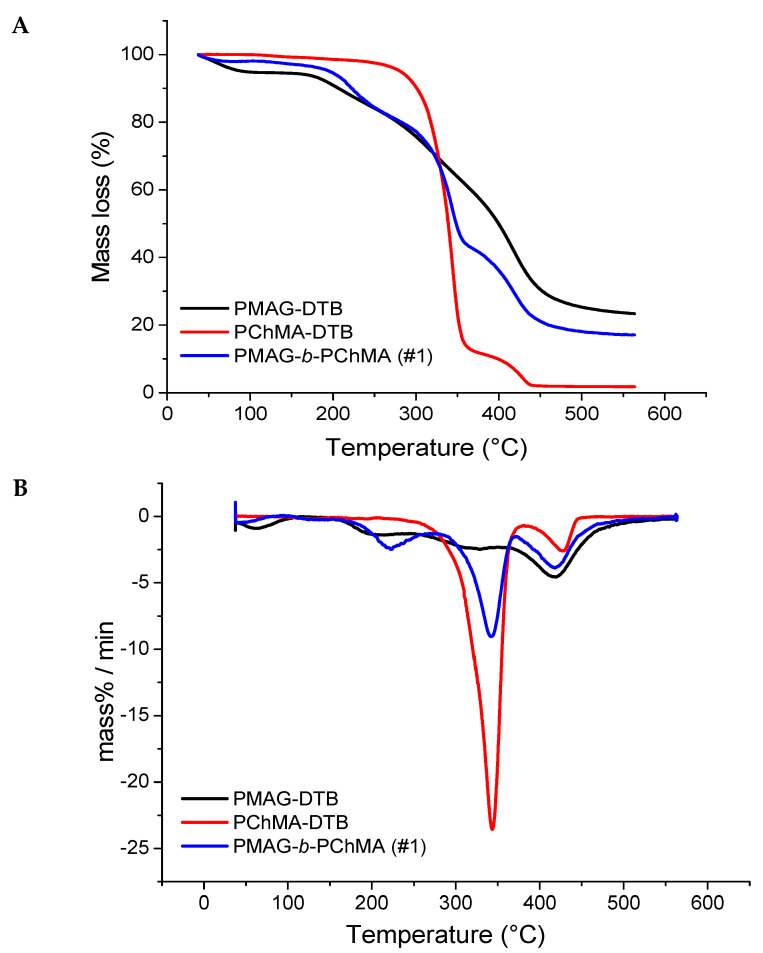
TGA (**A**) and DTG (**B**) plots for PMAG, PChMA, and their copolymer.

The investigation of the properties of novel polymers is very important for the understanding of their application capabilities. All block-copolymers obtained were analyzed by thermogravimetric analysis (TGA) to determine their thermal stability. Figure 6 shows the thermograms of homopolymers PMAG-DTB and PChMA-DTB, as well as their block-copolymer. As expected, the thermogram of PMAG-*b*-PChMA occupied an intermediate position between the thermograms of the parental homopolymers. 

Synthetic glycopolymers, such as polysaccharides, are highly hygroscopic compounds and are characterized by the initial mass loss on the thermogram up to 160 °C, which is associated with the evaporation of adsorbed water [62]. This process was observed for both PMAG-DTB and its block-copolymer with PChMA. The destruction of the homopolymer PMAG-DTB, similar to many other synthetic glycopolymers [62,63], proceeds in several steps. At first, the side chain of the carbohydrates is decomposed into water and carbon dioxide, and then the residual macromolecular chain is destroyed [63].

The destruction of the PChMA-DTB homopolymer is also of a stepwise manner. At first, the side chain is degraded with the cleavage of cholesterol groups up to 380 °C with a mass loss of approximately 88% (Figure 6). The stepwise nature of the decomposition of polymethacrylates with the cleavage of side groups at the first step is known [64]. Such destruction occurs up to 400 °C and is accompanied by the formation of poly(methacrylic anhydride). Further destruction of poly(methacrylic anhydride) can occur in various ways, both with the sequential degradation of the polymer chain, and with the cleavage of the side groups and the formation of a hydrocarbon backbone.

**Figure 7:**
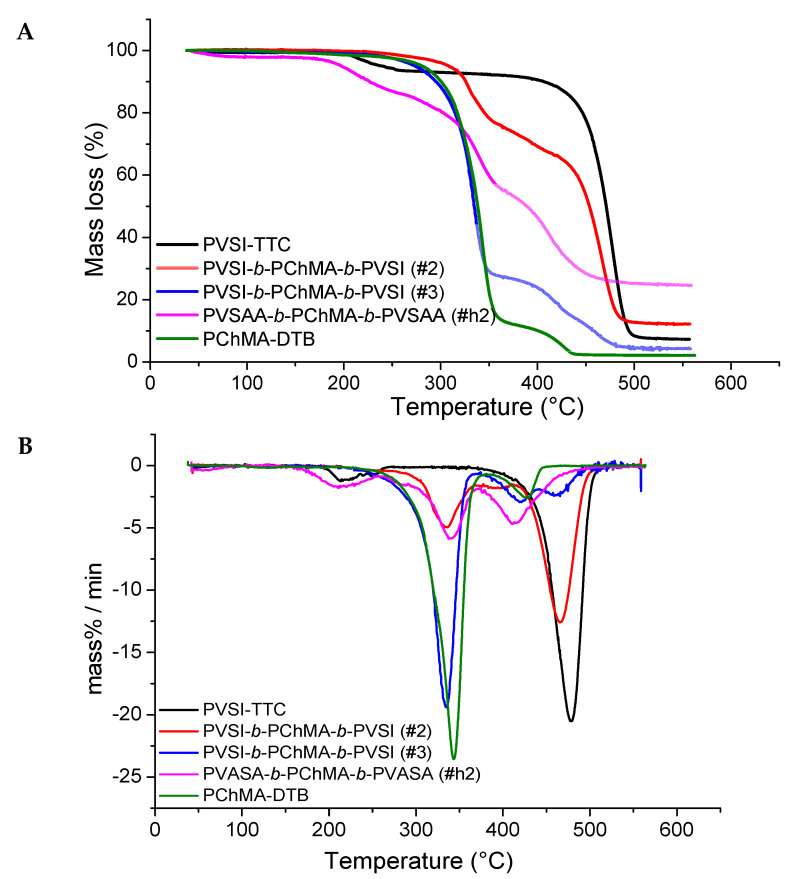
TGA (**A**) and DTG (**B**) plots for PVSI/PVSAA-containing copolymers with PChMA and corresponding homopolymers.

The analysis of thermograms of PVSI-containing block-copolymers and their homopolymers (Figure 7) showed an increase in thermal stability alongside an increase in the proportion of PVSI in the block-copolymer. Destruction of the PVSI homopolymer proceeds after 400 °C, and the mass loss of 7% up to 200 °C can be related to the solvent traces. Block-copolymers with different contents of PVSI and PChMA blocks show two stages of destruction on the thermograms. The temperatures of these processes appeared to be closer to those detected for the corresponding homopolymers for which the block-copolymers were enriched. Therefore, the thermogram for sample #2 (Table 1), which was enriched with PVSI fragment, was closer to the PVSI homopolymer. At the same time, sample #3, enriched with PChMA fragment (Table 1), had temperatures of mass loss closer to the PChMA homopolymer. The thermal stability of the amphiphilic block-copolymer was significantly reduced in comparison to the non-hydrolyzed PVSI-containing analog. The destruction of PVSAA-*b*-PChMA-*b*-PVSAA copolymers occurs in three main steps. The first step is responsible for the decomposition of the side chain of PVSAA, the second refers to the degradation of the side chain of PChMA, and finally, the third indicates the decomposition of the remaining polymer backbone.

Based on the data obtained, it can be concluded that sample #2 showed the highest thermal stability. It remained stable without significant mass loss up to ~300 °C, while its hydrolyzed analogue demonstrated thermal stability only up to ~200 °C. The amphiphilic PMAG-*b*-PChMA and PVSI-containing block-copolymer with a short PVSI fragment (sample #3) demonstrated only insignificant mass losses at temperatures of up to 200 °C, which is often connected with the evaporation of adsorbed solvent. 

### 2.4. Preparation and Characterization of Polymer Nanoparticles

**Figure 8:**
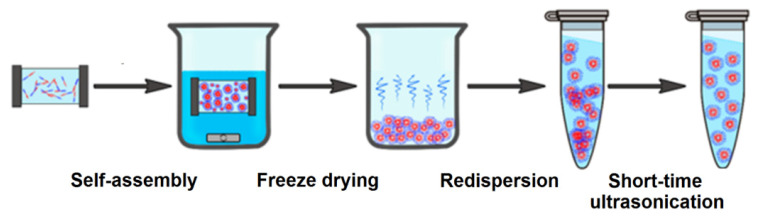
Scheme for the preparation of self-assembled soft nanoparticles.

Nanoparticles (NPs) were obtained due to the self-assembly of amphiphilic copolymers by the gradient phase inversion method during the replacing of an organic solvent (dimethyl sulfoxide (DMSO), DMF, etc.) with water (Figure 8). In this case, the replacement of the solvent leads to progressive aggregation of the polymer due to the loss of solubility and the formation of a colloid. For storage, the dispersion was subjected to soft drying by solvent sublimation (lyophilization). Before application, an aqueous solution of interest was added to the weighted sample and redispersion of NPs under short-term ultrasound exposure (30–60 s) was performed. 

**Figure 9:**
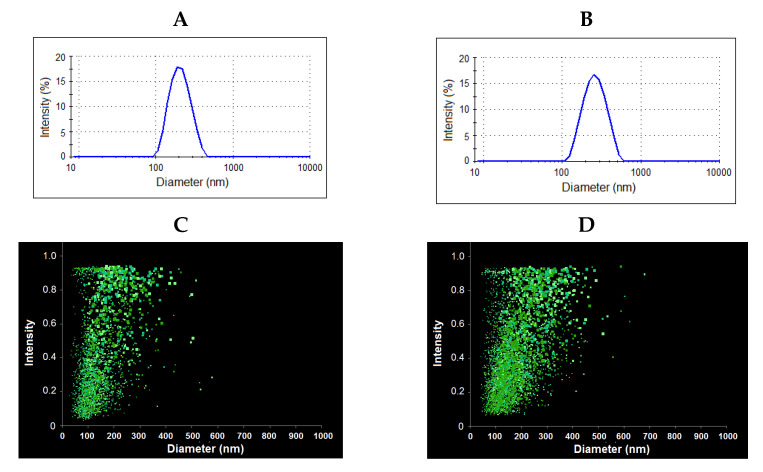
Results of dynamic light scattering (**A**,**B**) and nanoparticle tracking analysis (**C**,**D**) for PMAG-*b*-PChMA (**A**,**C**) and PVSAA-*b*-PChMA-*b*-PVSAA (**B**,**D**).

In all cases, the average hydrodynamic diameter (*D_H_*) and polydispersity index (PDI) were determined for nanoparticles lyophilized and redispersed in 0.01 M phosphate buffered saline (PBS) by dynamic light scattering (DLS) and nanoparticle tracking analysis (NTA). The results obtained are summarized in Table 2 and Figure 9. The results on the average hydrodynamic diameter obtained by both methods were in agreement with each other. 

The stability of the colloids is determined by gravitational and electrostatic components. The first component is connected to the size and weight of the particles, while the electrostatic repulsive forces are defined by the kinetic stability due to the magnitude of zeta potential. Thus, the smaller the particle size and the higher its surface charge, the more stable they are to aggregation. However, for colloidal solutions of high molecular weight substances, a stable suspension can exist even at zeta potential values of approximately ±20 mV [65]. In our case, all nanoparticles demonstrated the negative values of ζ-potential. For PMAG-*b*-PChMA, it was due to the ionization of the carboxylic group of R-end –C(CN)(CH_3_)–(CH_2_)_2_–COOˉ, while for PVSAA-based copolymers it was provided by the carboxyls of succinamic acid. The absolute value of ζ-potential increased with the elongation of the PVSAA chain (Table 2, samples #h2 and #h3).

**Figure 10:**
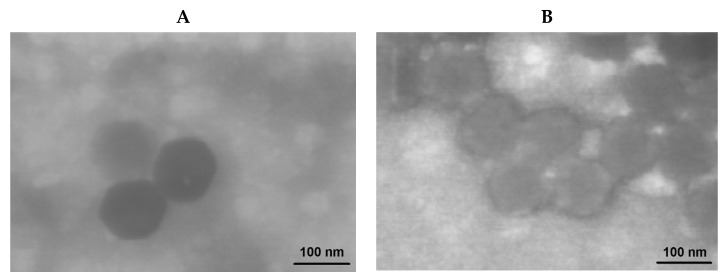
TEM images of PMAG-*b*-PChMA (**A**) and PVSAA-*b*-PChMA-*b*-PVSAA (sample #h2) (**B**) nanoparticles.

The morphology of nanoparticles was investigated using transmission electron microscopy (TEM) (Figure 10). In contrast to DLS and NTA providing the hydrodynamic diameter, TEM allows for the determination of the diameter of nanoparticles in a dry state. As expected, the hydrodynamic diameter of the nanoparticles under study was higher than the size determined in a dried state. This phenomenon is known for the soft materials, including micelles and polymersomes, from polymers of different nature [8,66,67,68]. The comparison of the images for nanoparticles obtained from PMAG-*b*-PChMA and PVSAA-*b*-PChMA-*b*-PVSAA makes it possible to distinguish some differences in their morphology. It is known from the literature that diblock-copolymers easily form micelles [69,70]. In turn, ABA copolymers, where A is a hydrophilic fragment and B is hydrophobic, are known to form nanoparticles of vesicular morphology [71]. Based on the literature data, it can be speculated that PMAG-*b*-PChMA and PVSAA-*b*-PChMA-*b*-PVSAA potentially form micelles and vesicles, respectively.

### 2.5. Cytotoxicity and Uptake by Macrophages

It is known that the size, shape, and aggregation of particles can affect the results of cellular experiments [72]. As the stability of nanoparticles in a protein-rich medium may differ from that in buffer solution, it was reasonable to evaluate the stability of obtained NPs in the culture medium before the cytotoxicity study [73]. To evaluate the stability of NPs, the PMAG-*b*-PChMA and PVSAA-*b*-PChMA-*b*-PVSAA colloids were incubated in 0.01 M PBS and Dulbecco’s Modified Eagle Medium (DMEM) containing 10% of fetal calf serum (FCS) at 37 °C. The monitoring of the hydrodynamic diameter of NPs by DLS within 14 days did not reveal any changes in average *D_H_* value for PVSAA-*b*-PChMA-*b*-PVSAA nanoparticles. PMAG-*b*-PChMA nanoparticles were also stable in buffer medium, while in DMEM-FCS they formed the 1–2 µm aggregates after 5 days of incubation. Potentially, the observed effect is associated with both the absence of sufficient surface charge and the affinity of some components of the culture medium to the saccharide residues of PMAG, which promote the adsorption of molecules from the culture medium on the surface of NPs. Thus, both types of nanoparticles were stable for 5 days, which is enough to study cytotoxicity uncomplicated by aggregation.

**Figure 11:**
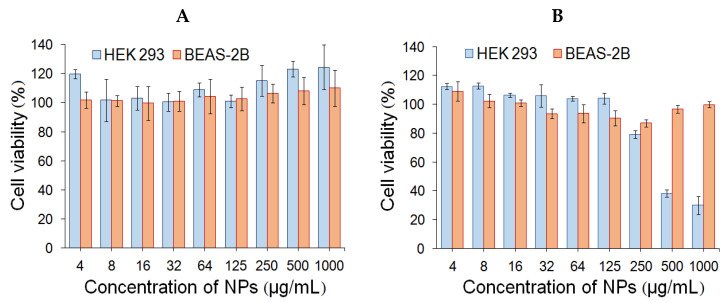
Cell viability of HEK293 and BEAS-2B cells during 24 h (**A**) and 72 h (**B**) for PVSAA-*b*-PChMA-*b*-PVSAA nanoparticles.

Human embryonic kidney cells (HEK293) and human lung epithelial cells (BEAS-2B) were used to evaluate cytotoxicity. The study of cell viability was carried out during the incubation of cells with NPs in the concentration range from 4 to 1000 µg/mL for 24 and 72 h. PMAG-*b*-PChMA NPs were non-toxic in the entire concentration range (IC_50_ ˃ 1000 µg/mL) for both cell lines (data not shown). Recently, the analogous results were also shown for NPs formed from other PMAG-containing block-copolymers [17,24]. In the case of PVSAA-*b*-PChMA-*b*-PVSAA NPs, the absence of cytotoxicity was observed upon incubation of NPs with BEAS-2B cells (IC_50_ ˃ 1000 µg/mL), while for HEK293 they showed cytotoxicity at concentrations above 250 µg/mL (IC_50_ ˃ 300 µg/mL) after 72 h (Figure 11). 

Besides cytotoxicity, the uptake by macrophages is another key property of NPs considered as drug delivery systems. Uptake by macrophages is one of the main mechanisms for the elimination of NPs in vivo. The rate of this process directly depends on the size, shape, and charge of nanoparticles [74]. It is known that the macrophage clearance of neutral and negatively charged NPs is slower than that of positively charged NPs [75]. 

In this study, flow cytometry was used for evaluation of the uptake by macrophages for the developed NPs. The analysis was performed after incubation of Cy5-labeled NPs for 6 h with mouse BALB/c monocyte macrophages (J774A.1 cell line). The labeling of NPs was carried out as described in Section 3.3.6. 

**Figure 12:**
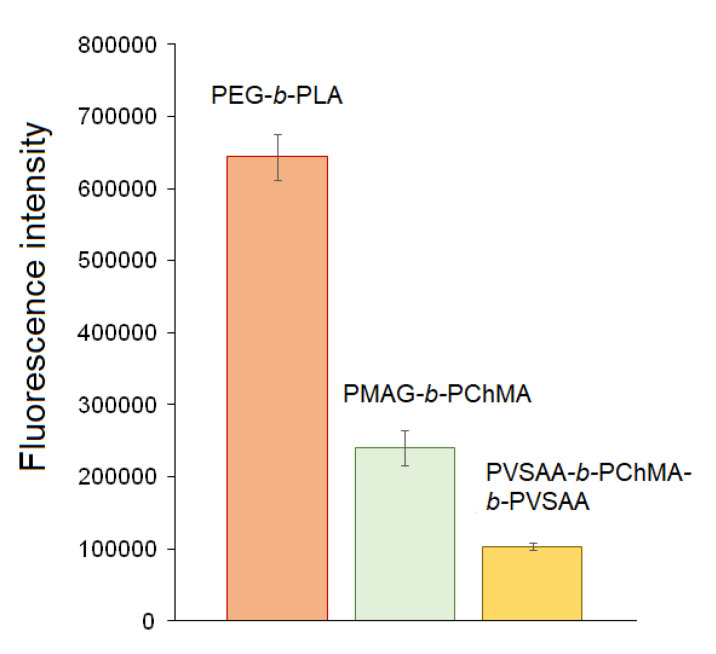
Uptake of the Cy-5 labeled nanoparticles by macrophages. In all cases, the amount of bound Cy5 was 3 µg/mg of NPs.

Currently, the modification of NPs with PEG is a widely used technique to protect nanoparticles from the fast uptake by macrophages [76]. In this regard, well-known PEG-*b*-PLA nanoparticles (D_H_ = 90 ± 16 nm) were used as a benchmark for comparison. Figure 12 shows that both PMAG-*b*-PChMA and PVSAA-*b*-PChMA-*b*-PVSAA NPs demonstrated the reduced uptake by macrophages in comparison with control PEGylated PLA nanoparticles. As expected, the lowest rate of uptake by macrophages was revealed for negatively charged PVSAA-based nanoparticles. 

Summarizing the results on cytotoxicity and uptake by macrophages, it can be deduced that both types of nanoparticles can be considered as promising drug delivery systems. 

### 2.6. Paclitaxel Delivery Systems 

Paclitaxel (PTX) is a hydrophobic cytostatic anti-cancer drug related to taxanes that has broad anti-cancer activity. In particular, PTX is effective in the treatment of ovarian, breast, lung, cervix, and pancreatic cancer [77]. For the encapsulation of PTX, a protocol recently developed for other amphiphilic block-copolymers was used in this study [2]. The encapsulation of PTX was carried out by dissolving the copolymer and the drug in DMSO, followed by freeze-drying the solution and further redispersion in 0.01 M PBS (pH 7.4). Using that approach, 50 and 100 µg of PTX was loaded per mg of nanoparticles. At the loading of 100 µg/mg of NPs, the hydrodynamic diameter (by DLS) of both kinds of nanoparticles was increased approximately twice.

**Figure 13:**
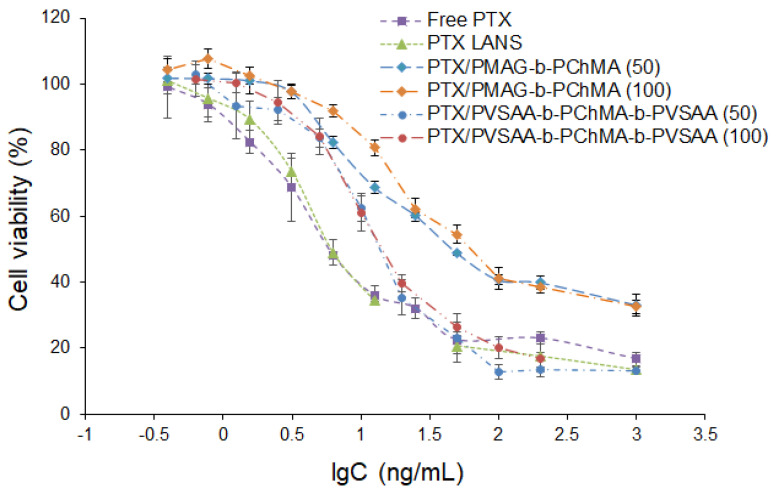
Dose–response curves for free PTX, commercial PTX LANS, and developed polymer nanoformulations (A549 cells, 72 h).

The biological activity of PTX formulations was studied using lung adenocarcinoma epithelial cells (A549 cell line). The PTX formulations were incubated with cells for 72 h in a concentration-dependent manner. Free PTX dissolved in DMSO and commercial formulation under the trade name Paclitaxel LANS^®^ (PTX LANS) were used for comparison as the controls. As seen from Figure 13, the application of the PVSAA-*b*-PChMA-*b*-PVSAA-based PTX formulation provided the most pronounced cell death than the PMAG-*b*-PChMA-based one. In particular, cell death at the maximal tested concentration was found to be 85% for the PVSSA-*b*-PChMA-*b*-PVSAA-based PTX formulation, and 68% for the PMAG-*b*-PChMA-based one. 

The IC_50_ values determined for tested formulations and free drug are summarized in Table 3. As expected, the lowest IC_50_ was detected for the free drug. The difference in IC_50_ for free and encapsulated PTX is a result of the different drug concentration in the medium during the incubation time. If the concentration for free drug remains constant over the experiment, it increases gradually for encapsulated forms while release is in progress. In the case of a PMAG-based PTX nanoformulation, the different PTX loading influenced the IC_50_ values; the increase in PTX loading contributed to the increase in IC_50._ This result may be due to the difference in the rate of release of the drug from the nanoparticles. In turn, for the PVSAA-based PTX nanoformulation, IC_50_ values did not depend on drug loading and were approximately two times higher than for free drug and commercial formulation. Similar results have been recently reported by Jiang et al. and Alani et al. for paclitaxel polymer formulations [78,79]. The differences in the release of PTX from PMAG and PVSAA-based nanoparticles may be due to both the different self-assembly of block-copolymers used and their interaction with cells. Despite the higher IC_50_ for PTX encapsulated forms, they may reduce side effects when administered systematically.

## 3. Materials and Methods

### 3.1. Materials

Monomers: MAG and ChMA were synthesized and purified using standard protocols described elsewhere [80,81]. 2-Hydroxyethylsuccinimide (95%) was purchased from Sigma-Aldrich (Darmstadt, Germany) and used as a precursor for the synthesis of VSI.

CTA agents and initiator: 4-cyanopentanoic acid-4-dithiobenzoate (DTB, >97%) and S,S-dibenzyl trithiocarbonate (TTC, >97%) were purchased from Sigma-Aldrich (Darmstadt, Germany) and used as received. 2,2’-Azobisisobutyronitrile (AIBN, 98%) (Acros Organics, Geel, Belgium) was purified by recrystallization from ethanol. 

Solvents: DMF, toluene, 1,4-dioxane, tetrahydrofuran (THF), chloroform, ethyl alcohol, and other solvents used in this work were purchased from Vecton Ltd. (St. Petersburg, Russia) and distilled by standard protocols before application.

Supplements: dialysis membranes with cut-off molecular weights (MWCO) of 1000, 2000, 3500, 8500 were the products of Orange Scientific (OrDialDClean regenerated cellulose dialysis tubing, Anaheim, CA, USA). Syringe filters with the hydrophilic and hydrophobic membranes MCE and PVDF-L, with a pore diameter of 0.22, 0.45, and 1.5 microns, were purchased from Filter-Bio (Jiangsu, China). 

Cell experiments: human embryonic kidney cells (HEK293), human lung epithelial cells (BEAS-2B), lung adenocarcinoma epithelial cells (A549), and mouse BALB/c monocyte macrophages (J774A.1) were purchased from CLS Cell Lines Service GmbH (Eppelheim, Germany). Dulbecco’s Modified Eagle Medium (Merck, Darmstadt, Germany), supplemented with 10% (*v*/*v*) fetal calf serum (FCS) (Biochrom, Berlin, Germany) and 1% (*v*/*v*) penicillin/streptomycin (P/S) (Biochrom, Berlin, Germany), were used for cell cultivation. CellTiter-Blue (CTB) cell viability assay reagent was purchased from Promega (Madison, WI, USA). MTT reagent was the product of ThermoFisher Scientific (Waltham, MA, USA).

Drugs: paclitaxel was purchased from Sigma-Aldrich (Darmstadt, Germany). Paclitaxel-LANS^®^, representing PTX solution in the mixture of Polyoxyl 35 castor oil and ethanol, was a product of Veropharm (Moscow, Russia). 

Other materials: PChMA, used for comparison in TGA, had the following characteristics (SEC): *M_w_* = 8900, *Đ* = 1.8. PEG-*b*-PLA nanoparticles (*D_H_* = 90 ± 16 nm) were used as control in experiments on uptake by macrophages which were obtained as described in our recent work [21]. Cy5-NH_2_ dye was purchased from Lumiprobe (Moscow, Russia). *N*-hydroxysuccinimide (NHS) and 1-ethyl-3-(3-dimethylaminopropyl)carbodiimide (EDC) were purchased from Sigma-Aldrich (Darmstadt, Germany). 

All buffer solutions were prepared from analytically pure reagents and filtered through Millipore membrane filters with a pore size of 0.45 microns (Merck, USA). During the cell experiments, all solutions and buffers were pre-autoclaved, after which they were stored at 4 °C. 

### 3.2. Instruments for Polymer Characterization

^1^H NMR spectra were recorded on a Bruker AC-400 (400.1 MHz) (Karlsruhe, Germany) relative to the tetramethylsilane signal in a solvent being either CDCl_3_ (7.24 ppm) or D_2_O (4.27 ppm (25 °C), 4.4 ppm (60 °C)). IR spectra of polymers were recorded on the IR Fourier spectrometer IRAffinity-1 of Shimadzu (Kyoto, Japan) in KBr tablets. 

The determination of the molecular weights and dispersity (*Đ*) of homopolymers was carried out by size-exclusion chromatography using an Agilent-1260 Infinity liquid chromatograph (Santa Clara, CA, USA) equipped with refractometric, light-scattering, and viscometric detectors in combinations with two Agilent PLgel MIXED-C columns (7.5 × 300 mm, 5 µm). SEC analysis was performed in DMF containing 0.1 mol/L LiBr at 50 °C for PMAG, and in THF at 40 °C for PVSI. 

Thermogravimetric analysis was carried out on TG209 F1 Libra (Netzsch, Germany) thermomicroscales with an automatic sample feeding system in a nitrogen atmosphere at a constant heating rate of 10 °C/min. The range of the studied temperatures varied from 40 to 600 °C. Preliminary sample preparation consisted of grinding and homogenization of copolymer samples. 

### 3.3. Methods

#### 3.3.1. Synthesis and Characterization of PMAG-*b*-PChMA

The synthesis of PMAG-*b*-PChMA was performed by RAFT polymerization using PMAG-DTB as a macro-RAFT agent. The protocol for the PMAG-DTB synthesis was developed earlier and included the use of 4-cyanopentanoic acid-4-dithiobenzoate (DTB) chain-transfer agent (CTA) [24]. Briefly, it was synthesized by RAFT polymerization in DMF using the following molar ratio of components: [MAG]:[DTB]:[AIBN] = 20:1:0.25. The polymerization process was carried out in a Schlenk tube at 70 °C with continuous stirring for 16 h. Other details on the PMAG-DTB synthesis and characterization can be found in our recent papers [17,24]. For ^1^H NMR spectroscopy (D_2_O) (δ, ppm) of PMAG, -–CH_3_: 0.93–1.39; –CH_2_–C–: 1.39–2.11; glucose ring: 3.4–4.0; –C_1_H_1_ α: 5.03–5.30 and β: 4.78; DTB: R group –CH_2_–CH_2_–COOH: 2.55; –CH_2_–CH_2_–COOH: 1.88, 1.98; aromatic protons of Z groups at *o*-, *p*-, and *m*-positions, respectively: 7.52, 7.66, and 7.88. The obtained PMAG-DTB characterized conversion was 76% and *M_n_* = 4600, *Đ* = 1.05.

For the synthesis of PMAG-*b*-PChMA, a polymerization mixture was prepared by dissolving 0.1045 g (0.023 mmol) PMAG-DTB, 0.1903 g (0.419 mmol) ChMA, and 0.00137 g (0.008 mmol) AIBN in a mixture of solvents consisting of 2.5 mL DMF, 2.2 mL toluene, and 0.2 mL chloroform. As a result, the following molar ratio of components were used for polymerization: [PMAG-DTB]:[ChMA]:[AIBN] = 1:18:0.35. Before the polymerization, the reaction mixture placed into the Schlenk tube was degassed via four freeze-evacuated-thaw cycles, refilled with argon, and then immersed in a preheated oil bath. The polymerization process was carried out at a temperature of 60 °C with intensive stirring for 40 h. The product was precipitated in 50 mL of diethyl ether, then centrifuged, separated by decantation, and dried in a desiccator. The purification of the block-copolymer from macroinitiator and low-molecular impurities was carried out by dialysis (MWCO = 8500) against DMF, DMF/water = 50/50 (*v*/*v*), and water. The product was lyophilized and stored at 4 °C. The product yield was 48%. For FTIR spectroscopy (cm^−1^), CONHR: 3369 (NH_st_), 1640 (C=O_st_, «amide I»), and 1533 (NH_δ_, «amide II»); C–O–H: 1303 (C–O–H_δ_), 1033 (C–O_st_); CH_2_: 2939 (ν_as_); CH_3_: 2870 (ν_s_), 1468 (δ_as_), and 1383 (δ_s_); CH_2_–COOR: 1728 (C=O_st_); R’-COOR: 1259 (CO–O_st as_) and 1145 (O–C–C_st as_).

The content of PChMA in the PMAG-*b*-PChMA was determined by FTIR spectroscopy using a calibration plot built for a set of mixtures of PMAG and PChMA homopolymers taken in different ratios (Figure S2, Supplementary Materials). The intensities of characteristics bands at 1728 cm^−1^ for PChMA (C=O valence vibrations in esters) and at 1534 cm^−1^ for PMAG (“amide II”) were selected for analysis. The calculation of the number average molecular weight of the hydrophobic fragment in the block-copolymer was carried out by determining the molar fraction of PChMA according to the calibration plot and by considering the number average molecular weight of the hydrophilic block of PMAG, determined by the SEC method with triple detection.

#### 3.3.2. Synthesis of PVSI-*b*-PChMA-*b*-PVSI 

The synthesis of VSI was carried out in two steps. First, the acetylation of 2-hydroxyethylsuccinimide was carried out via reaction of 2-hydroxyethylsuccinimide (1 mol) and acetic anhydride (1.5 mol) at 150–155 °C for 6 h. Then, acetic acid was distilled from the resulting mixture under vacuum. Second, pyrolysis of 2-acetoxyethylsuccinimide was carried out in a pyrolysis furnace at 600 °C. The pyrolyzate represented a dark-brown liquid, from which VSI was distilled at 100–120 °C (133–400 Pa). VSI is a light-yellow solid compound under room temperature. The purification of VSI was carried out by recrystallization from isopropyl alcohol. The yield was 46%. ^1^H NMR (DMSO-d6) (δ, ppm): 2.66 (4H, s), 5.02 (1H, d), 5.95 (1H, d), and 6.64 (1H, dd). The melting point was 48.5 °C.

As in previous cases, the synthesis of PVSI-*b*-PChMA-*b*-PVSI copolymers was carried out by RAFT polymerization. First, PVSI was synthesized using VSI, *S*,*S*-dibenzyl trithiocarbonate, and AIBN as monomer, CTA, and initiator, respectively, according to the previously developed protocol [82,83]. Briefly, 25% solution of VSI in dioxane was placed into the Schlenk tube, degassed via four freeze-evacuated-thaw cycles, refilled with argon, and then polymerized at 80 °C for 7 h. The loading volume was 3–4 mL. The following ratios were used for polymerization: [CTA]:[VSI] = 3.3 × 10^−2^ and 10^−1^ (Table 1, samples #2 and #3, respectively), and [AIBN]:[VSI]=10^−3^. After synthesis, the polymer was precipitated into a 10-fold excess of distilled water, filtered, and then repeatedly washed with distilled water until the unreacted monomer was completely removed (the solubility of the VSI in water at 20 °C is approximately 5 *wt*%). The absence of VSI in the polymer was estimated by the absence of characteristic signals of vinyl protons in the ^1^H NMR spectra. The samples were dried in vacuum at 60 °C up to a constant mass. 

The ratio between macro-RAFT agent, monomer, and initiator was constant in all cases and was the following: [PVSI-TTC]:[ChMA]:[AIBN] = 1:20:0.4. For the synthesis of PVSI-*b*-PChMA-*b*-PVSI, a polymerization mixture was prepared by the dissolving of 0.013 mmol PVSI-TTC, 0.265 mmol ChMA, and 0.005 mmol AIBN in a mixture consisting of 0.8 mL DMF, 0.4 mL toluene, and 0.4 mL dioxane. Before polymerization, the reaction mixture in the Schlenk tube experienced four freeze-evacuated-thaw cycles, was refilled with argon, and then incubated at 80 °C under intensive mixing for 20 h. After that, the reaction mixture was cooled at −18 °C for 30 min, then dissolved in a small portion of chloroform and precipitated in 50 mL of ethyl alcohol. The precipitate was separated by decantation and dried in a desiccator. The yield of copolymer was 60%. For ^1^H NMR (CDCl_3_) (δ, ppm), PChMA fragment: 5.3–5.5 (s, C=C–H), 0.7 (s, C–CH_3_), and 0.8 (*d*–C–CH_3_); PVSI fragment: 3.3–3.5 (s, H–C–N); DTB: 7.0–7.3 (m, H–Ar). For IR spectroscopy (cm^−1^), CONRCO: 1700 (C=O st); CH_2_: 2942 (ν_as_), 2868 (ν_s_), and 1435 (δ, –CH_2_); CH_3_: 1385 (δ_s_); RR’C=CR’’H: 820 (δ_CH_); R’-COOR: 1258 (ν_C–O_). 

The number of monomer units in PVSI was calculated based on the number average molecular weight determined by the SEC. The number of PChMA units was determined as a ratio of the integral signal intensities in the ^1^H NMR spectra at 5.3–5.4 and 3.3–3.5 ppm, which are characteristic for the protons in H–C=C group of PChMA and H–C–N group in PVSI, respectively. 

#### 3.3.3. Preparation of PVSAA-*b*-PChMA-*b*-PVSAA

To obtain amphiphilic copolymers, the PVSI block in PVSI-*b*-PChMA-*b*-PVSI was subjected to alkaline hydrolysis under mild conditions. Exactly 1.33 mL of 2.5% aqueous NaOH solution was added to a solution of 0.1608 g (0.006 mmol) PVSI-*b*-PChMA-*b*-PVSI in a mixture of 12 mL of dioxane and 7 mL of toluene. The reaction mixture was left under stirring at 22 °C for 4 h, and then neutralized by 0.1 M HCl solution. Then, a small amount of DMF/toluene mixture was added to the reaction medium and the product was purified by dialysis (MWCO = 3500) against DMF, DMF/water = 50/50 (*v*/*v*), and water, respectively. The product obtained was lyophilized and stored at 4 °C. For FTIR spectroscopy (cm^−1^), CH_2_COOH: 1725 (C=O_st_), 3086 (O–H_st_), and 907 (O–Hδ); CONHR: 3300 (NHst), 1654 (C=O_st_, «amide I»), and 1555 (NH_δ_, «amide II»); CH_2_: 2942 (ν_as_), 2868 (ν_s_), and 1435 (δ, CH_2_); CH_3_: 1382 (δ_s_); RR’C=CR’’H: 820 (δ_CH_); R’–COOR: 1181c (O–C–C_st as_) and 1256 (ν_C–O_). 

#### 3.3.4. Preparation and Characterization of Nanoparticles 

Nanoparticles (NPs) were prepared as a result of the self-assembly of amphiphilic block-copolymers by a gradient phase inversion method [68]. For this, the copolymers were dissolved in DMSO and slowly transferred into water by dialysis. Self-assembled particles were then freeze-dried and stored at 4 °C. Before use, the dispersion of NPs was prepared by redispersion of dried polymer particles for 30–60 s under sonication by means of the ultrasonic probe (UP 50H Hielscher Ultrasonics, Teltow, Germany) in water or 0.01 M phosphate buffered saline (PBS), pH = 7.4. 

All characteristics of the nanoparticles were determined for colloids obtained after lyophilization and redispersion. Hydrodynamic diameter (*D_H_*), PDI, and ζ-potential of the nanoparticles were determined by dynamic light and electrophoretic scattering (DLS and ELS, respectively) using a Zetasizer Nano-ZS Malvern (Malvern, UK) equipped with a He–Ne laser at 633 nm at a scattering angle of 173° and 25 °C. Additionally, hydrodynamic diameter was determined by nanoparticle tracking analysis (NTA) Malvern Nanosight NS300 (Malvern, UK). The particle morphology was studied by transmission electron microscopy (TEM) using a Jeol JEM-2100 HC (Tokyo, Japan). Before analysis, a few drops of a colloid were placed onto a copper grid covered by carbon, and dried. Then, the grid was stained with 1% (*w*/*v*) uranyl acetate solution for 30–60 s and was used for analysis.

To study the stability of NPs, the colloids of polymer nanoparticles were prepared by dispersing 1 mg of the particles in 1 mL of 0.01 M PBS (pH =7.4) under sonication for 30–60 s. The solution was diluted three times with PBS or DMEM-FCS and incubated at 37 °C for 14 days. The changes in hydrodynamic diameter were monitored by DLS. 

#### 3.3.5. Encapsulation of Paclitaxel 

PTX was encapsulated using the previously developed protocol [17]. The difference was that here we used 50 or 100 μL of a solution of PTX in DMSO (1.0 mg/mL) for loading per 1 mg of polymer. Other details remained unchanged. 

#### 3.3.6. Uptake by Macrophages 

To study uptake by macrophages using flow cytometry, the particles were modified with Cy5–NH_2_ label. Nanoparticles were labeled via the reaction of activated ester or aldehyde groups with the amino group of Cy5. In particular, PVSAA-*b*-PChMA-*b*-PVSAA and PEG-*b*-PLA, both bearing carboxylic groups, were activated by EDC and NHS to form activated NHS esters, as previously described [21]. PMAG was oxidized with NaIO_4_ to form reactive aldehyde groups, as described elsewhere [25]. The amount of bound Cy5 was 3 µg/mg of NPs in all cases. 

Mouse BALB/c monocyte macrophage (J774A.1 cell line) was used to study the uptake of nanoparticles. The cells were cultivated in DMEM supplemented with 10% (*v*/*v*) fetal calf serum (FCS) and 1% (*v*/*v*) penicillin/streptomycin (P/S) in a humidified environment at 37 °C/5% CO_2_. The 2 × 10^6^ cells in DMEM medium were cultivated in 2 mL of medium in 6-well plates for 24 h in a humidified environment at 37 °C and 5% CO_2_. Then, the medium was removed and replaced with 1 mL of medium containing Cy5-labeled NPs (1 µg/mL). The plates were exposed at 37 °C and 5% CO_2_ for 6 h. After cultivation and exposure to NPs, the cells were washed twice with warm PBS, detached by cell lifter, and resuspended in 500 µL PBS. After that, fluorescence signals were measured via flow cytometry. The samples were analyzed by a BD Accuri C6 ThermoFisher (Waltham, MA, USA) with a 640 nm laser. Cy5 fluorescence was collected by a 670 LP band-pass filter. At least 30,000 events per sample were analyzed. Only viable cells were involved in the analysis.

#### 3.3.7. Cytotoxicity Study

Cytotoxicity of empty and paclitaxel (PTX)-loaded NPs were examined in a concentration-depended manner. The concentration of empty nanoparticles varied from 4 to 1000 µg/mL. The PTX and PTX-loaded NP cytotoxicity was tested in a range from 0.625 to 1000 ng/mL. HEK293 and BEAS-2B cell lines were used to evaluate the cytotoxicity of empty NPs and A549 to determine the IC_50_ for PTX and its polymer formulations. The cells were cultivated in a humidified environment at 37 °C/5% CO_2_. The medium was changed two times per week. Before reaching confluence, the cells were subcultivated using trypsin.

Moreover, 8 × 10^3^ cells per well were seeded in a 96-well plate (100 μL/well) and cultivated under a humidified atmosphere of 5% CO_2_ at 37 °C for 24 h. After that, the medium was replaced by the tested samples of different concentrations in the culture medium. Cell viability was determined after 24 or 72 h treatment using the CTB assay for empty NPs [17] and MTT assay for PTX and its polymer formulations. In the latter case, the medium was removed, MTT solution (5 mg/mL in PBS) was added into each well and the plate was incubated for 2–4 h at 37 °C in a CO_2_ incubator until formazan crystals formed. After that, 50 µL of DMSO was added into each well, including control ones. The absorbance was measured at 570 nm in a multiwell plate reader (ThermoFisher Multiscan Labsystems, Waltham, MA, USA). A non-linear fitting/growth/sigmoidal/dose–response curve fitting function in OriginPro 8.6 was used to calculate half-maximal inhibition concentrations (IC_50_). The data from the biological experiments are presented as mean values ± SD (*n* = 4). 

## 4. Conclusions

Two amphiphilic block-copolymers combining the biomimetic properties of natural macromolecules and characterized by greater stability to biodegradation were synthesized in this study. The introduction of PChMA and an increase in its ratio in the copolymer contributed to the growth in thermal stability. The obtained copolymers tended to self-assemble into nanoparticles with a hydrodynamic diameter close to 200 nm. Nanoparticles based on a copolymer containing PVSAA with a high negative charge were stable in both the buffer and the culture medium for two weeks, while the copolymer containing PMAG aggregated after five days of incubation in the culture medium. The cytotoxicity study did not reveal any toxicity for PMAG-bearing nanoparticles, while PVSAA-based nanoparticles were non-toxic at concentrations of up to 250 mg/mL for 72 h. Both kinds of nanoparticles demonstrated a low uptake by macrophages compared to the widely used PEG-*b*-PLA nanoparticles. Furthermore, nanoparticles were able to encapsulate hydrophobic anti-cancer drug paclitaxel with high efficiency. The encapsulated drug retained its biological activity against specific tumor cells. In general, the developed polymer NPs, based on bio-inspired block-copolymers with a non-biodegradable main chain, demonstrated high potential as drug delivery systems for the hydrophobic anti-tumor drug paclitaxel. 

## Figures and Tables

**Table 1:** The molecular weight characteristics and polymerization degree of amphiphilic block-copolymers.

Sample	Hydrophilic Block ^a^				Hydrophobic Block	
	*M_n_*	*M_w_*	*Đ*	*DP*	*DP*	*M_n_*
	**PMAG**				**PChMA ^b^**	
#1	4600	4800	1.05	18	18	8100
	**PVSI**				**PChMA ^c^**	
#2	14,300	19,600	1.37	114	27	12,150
#3	2800	3000	1.10	22	43	19,350

*^a^* Size-exclusion chromatography; *^b^* FTIR spectroscopy; *^c^* ^1^H NMR spectroscopy; Designations: *M_n_*—number average molecular weight; *M_w_*—weight average molecular weight; *Đ*—dispersity; *DP*—degree of polymerization.

**Table 2:** Characteristics of the nanoparticles self-assembled from amphiphilic block-copolymers.

Block-Copolymer	*D_H_* (nm)		PDI	ζ-Potential (mV)
	DLS	NTA		
PMAG-*b*-PChMA (#1)	203 ± 65	191 ± 75	0.16	−14.5 ± 1.7
PVSAA-*b*-PChMA-*b*-PVSAA (#h2)	230 ± 90	219 ± 79	0.20	−66.2 ± 2.1
PVSAA-*b*-PChMA-*b*-PVSAA (#h3)	220 ± 85	–	0.20	−19.0 ± 0.1

**Table 3:** IC_50_ values determined on A549 cells for the free PTX, Paclitaxel LANS^®^, and developed PTX formulations.

Formulation	Loading (µg/mg of NPs)	IC_50_ (ng/mL)
Free PTX	−	4.4 ± 0.4
PTX LANS	−	4.9 ± 0.3
PTX loaded in PMAG-*b*-PChMA NPs	50	16.6 ± 1.0
PTX loaded in PMAG-*b*-PChMA NPs	100	25.1 ± 0.9
PTX loaded in PVSAA-*b*-PChMA-*b*-PVSAA NPs	50	10.6 ± 0.5
PTX loaded in PVSAA-*b*-PChMA-*b*-PVSAA NPs	100	11.3 ± 1.0

## Data Availability

The data presented in this study are available on request from the corresponding author.

## Supplementary Materials

The following are available online at https://www.mdpi.com/article/10.3390/ijms222111457/s1. Figure S1: ¬^1^H NMR spectrum (400 MHz, D_2_O, 25 °C) of PMAG-DTB; Figure S2: Calibration plot built for the mixture of PMAG and PChMA homopolymers and used for the determination of PChMA content in the PMAG-*b*-PChMA; Figure S3: ^1^H NMR spectrum (400 MHz, CDCl_3_, 25 °C) of block-copolymer PVSI-*b*-PChMA-*b*-PVSI (#2).
