# Supplementary material for: Self-Assembled Nanoparticles Based on Block-Copolymers of Poly(2-Deoxy-2-methacrylamido-d-glucose)/Poly(N-Vinyl Succinamic Acid) with Poly(O-Cholesteryl Methacrylate) for Delivery of Hydrophobic Drugs
Source: Int J Mol Sci. 2021 Oct 24;22(21):11457. doi: 10.3390/ijms222111457 (PMC8583880; doi:10.3390/ijms222111457)
Supplement: Supplementary file 1 [file ijms-22-11457-s001.zip › ijms-1416897-supplementary.pdf]

International Journal of
Molecular Sciences

MDPI

Supplementary Materials

Self-Assembled Nanoparticles Based on Block-Copolymers of Poly(2-deoxy-2-methacrylamido-D-glucose)/Poly(*N*-vinyl succinamic acid) with Poly(*O*-cholesteryl methacrylate) for Delivery of Hydrophobic Drugs

Mariia Levit [1], Alena Vdovchenko [2], Apollinariia Dzhuzha [1,2], Natalia Zashikhina [1], Elena Katernyuk [1,2], Alexey Gostev [3], Eugene Sivtsov [1,3], Antonina Lavrentieva [4], Tatiana Tennikova [2] and Evgenia Korzhikova-Vlakh [1,*]

[1] Institute of Macromolecular Compounds, Russian Academy of Sciences, Bolshoy pr. 31, 199004 St. Petersburg, Russia
[2] Institute of Chemistry, Saint-Petersburg State University, Universitetskiy pr. 26, 198504 St. Petersburg, Russia
[3] Saint-Petersburg State Institute of Technology, Technical University, Moskovskiy pr. 26, 190013 St. Petersburg, Russia
[4] Institute of Technical Chemistry, Gottfried-Wilhelm-Leibniz University of Hannover, 30167 Hannover, Germany
* Correspondence: vlakh@hq.macro.ru

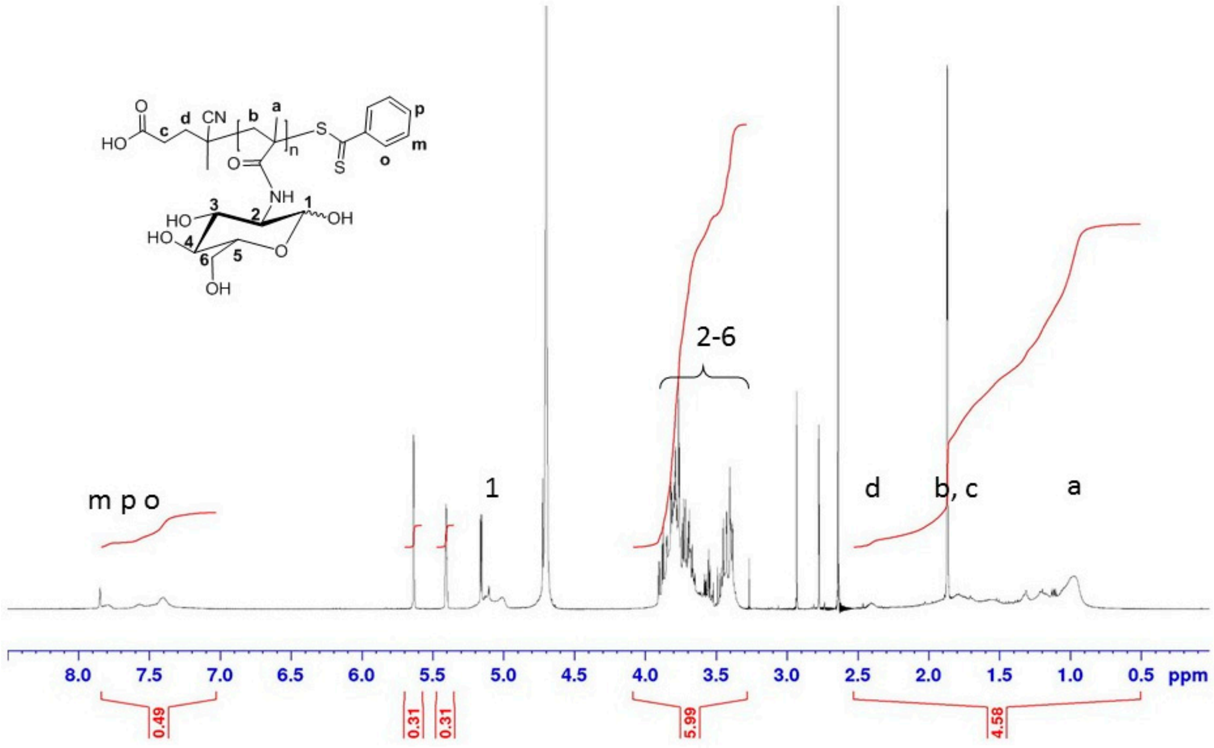

Figure S1. ^{1}H NMR spectrum (400 MHz, D_2O, 25 °C) of PMAG-DTB.

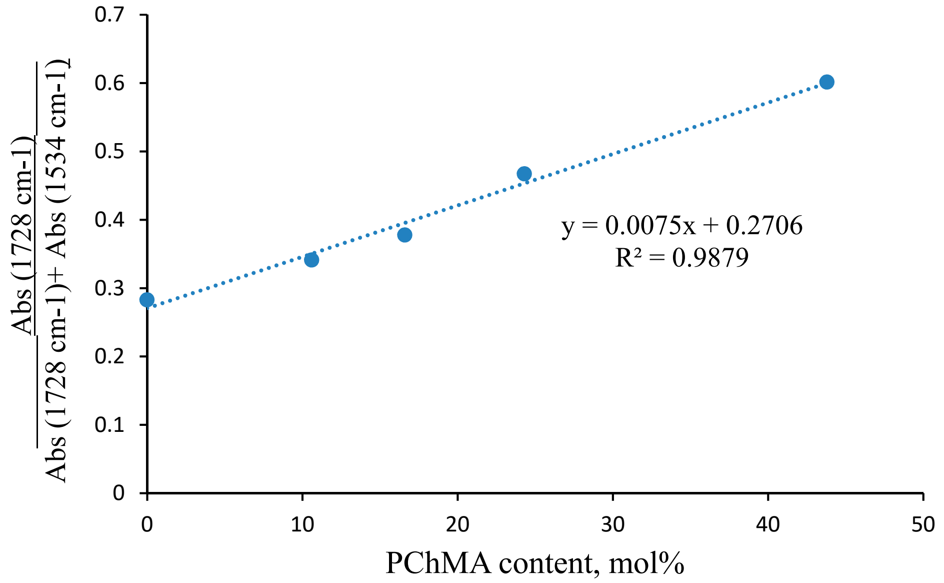

Figure S2. Calibration plot built for the mixture of PMAG and PChMA homopolymers, and used for the determination of PChMA content in the PMAG-*b*-PChMA.

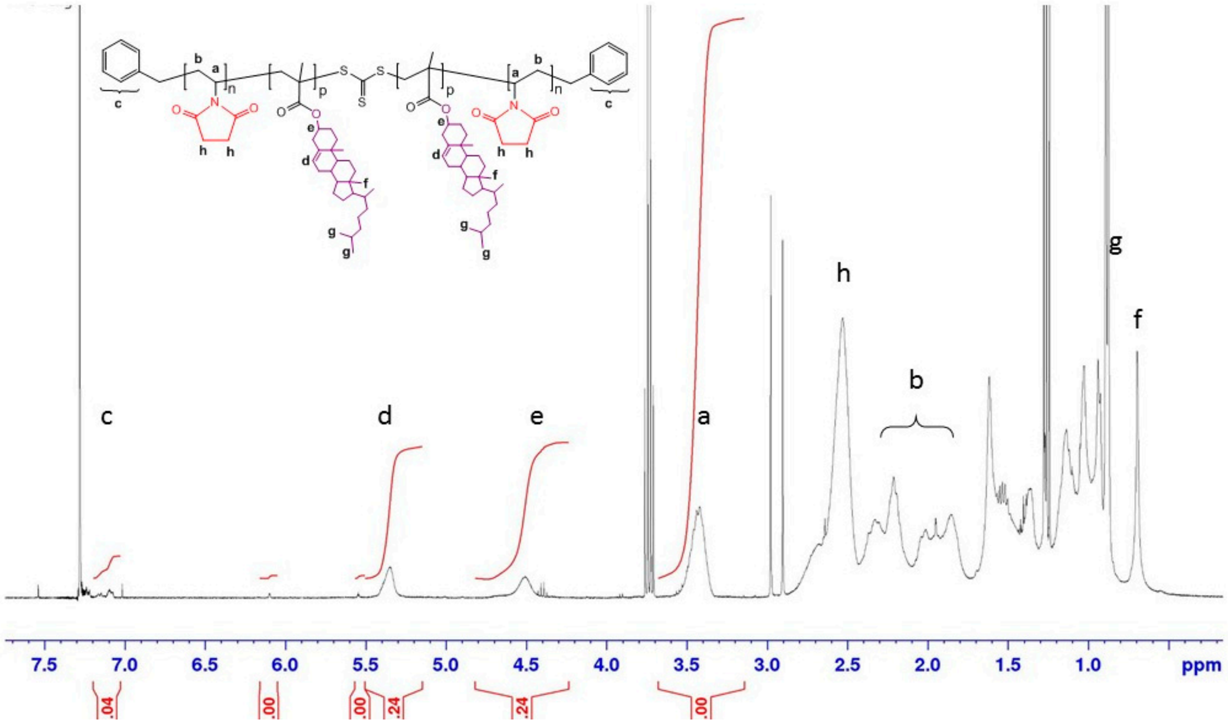

Figure S3. ^{1}H NMR spectrum (400 MHz, $CDCl_3$, 25 °C) of block-copolymer PVSI-*b*-PChMA-*b*-PVSI (#2).
